# Investigation of parenteral nutrition-induced hepatotoxicity using human liver spheroid co-cultures

**DOI:** 10.1007/s00204-024-03773-8

**Published:** 2024-05-14

**Authors:** Milos Mihajlovic, Sybren De Boever, Andrés Tabernilla, Ellen Callewaert, Julen Sanz-Serrano, Anouk Verhoeven, Amy Maerten, Zenzi Rosseel, Elisabeth De Waele, Mathieu Vinken

**Affiliations:** 1 Department of Pharmaceutical and Pharmacological Sciences, Vrije Universiteit Brussel, Laarbeeklaan 103, 1090 Brussels, Belgium; 2 Department of Pharmacy, Universitair Ziekenhuis Brussel (UZ Brussel), Brussels, Belgium; 3 Department of Clinical Nutrition, Universitair Ziekenhuis Brussel (UZ Brussel), Brussels, Belgium; 4 Faculty of Medicine and Pharmacy, Vrije Universiteit Brussel, Brussels, Belgium

**Keywords:** Total parenteral nutrition, Intestinal failure-associated liver disease, Liver spheroid co-culture, RNA sequencing, Liver toxicity

## Abstract

Parenteral nutrition (PN) is typically administered to individuals with gastrointestinal dysfunction, a contraindication for enteral feeding, and a need for nutritional therapy. When PN is the only energy source in patients, it is defined as total parenteral nutrition (TPN). TPN is a life-saving approach for different patient populations, both in infants and adults. However, despite numerous benefits, TPN can cause adverse effects, including metabolic disorders and liver injury. TPN-associated liver injury, known as intestinal failure-associated liver disease (IFALD), represents a significant problem affecting up to 90% of individuals receiving TPN. IFALD pathogenesis is complex, depending on the TPN components as well as on the patient’s medical conditions. Despite numerous animal studies and clinical observations, the molecular mechanisms driving IFALD remain largely unknown. The present study was set up to elucidate the mechanisms underlying IFALD. For this purpose, human liver spheroid co-cultures were treated with a TPN mixture, followed by RNA sequencing analysis. Subsequently, following exposure to TPN and its single nutritional components, several key events of liver injury, including mitochondrial dysfunction, endoplasmic reticulum stress, oxidative stress, apoptosis, and lipid accumulation (steatosis), were studied using various techniques. It was found that prolonged exposure to TPN substantially changes the transcriptome profile of liver spheroids and affects multiple metabolic and signaling pathways contributing to liver injury. Moreover, TPN and its main components, especially lipid emulsion, induce changes in all key events measured and trigger steatosis.

**Supplementary Information:**

The online version contains supplementary material available at 10.1007/s00204-024-03773-8.

## Introduction

Parenteral nutrition (PN) is a form of artificial nutrition serving as a life-saving approach for various malnourishment-associated conditions and diseases. PN consists of a mixture of lipid emulsions, carbohydrates, amino acids, vitamins, minerals, electrolytes, and trace elements, which is delivered intravenously to patients with impaired gastrointestinal function and/or not suitable for enteral feeding (Berlana 2022). Indications for PN include gastrointestinal obstruction and motility disorders, short bowel syndrome, malignancies, hypercatabolic states, as well as premature birth, among others (Berger and Pichard 2022). PN can be administered via a smaller peripheral vein (peripheral PN) or through a large central vein (central PN) and can be applied as a short-term as well as a long-term treatment (Berlana 2022). When intravenously administered PN is the only source of energy, it is defined as total parenteral nutrition (TPN), as opposed to supplemental parenteral nutrition indicating a combination of intravenous nutrition with oral or enteral nutrition used to avoid energy deficits (Berger et al. 2022; Berlana 2022). Despite the evident benefits, TPN treatment can cause health complications with potentially fatal outcomes. Besides problems related to venous access and catheter site infections, distinct adverse effects linked to the nutrition and metabolic conditions of the patients can manifest, including hyperglycemia, dehydration, electrolyte imbalance, thrombosis, encephalopathy, bone disorders, and liver injury (Sobotka and Camilo 2009; Berlana 2022). The latter, which is commonly known as PN-associated liver disease or intestinal failure-associated liver disease (IFALD), is one of such complications occurring during TPN treatment (Lal et al. 2018). In the context of IFALD, liver injury is usually manifested as steatosis, cholestasis, or a combination of both conditions. The incidence of IFALD in patients receiving TPN can be as high as 90%, depending on the underlying conditions (Kumpf 2006; Lakananurak and Tienchai 2019; Bischoff et al. 2020; Weylandt et al. 2023). In extreme cases of long-term TPN, end-stage liver disease might occur with a rather high mortality rate of up to 34% within 4 years (Chan et al. 1999; Cavicchi et al. 2000). The main risk factors linked to disease development are patient related (i.e., lack of enteral feeding, gut dysbiosis, and infections) and PN related (i.e*.,* caloric overload, intravenous lipid emulsion composition, and (micro)nutrient deficiency or excessive administration) (Lakananurak and Tienchai 2019; Lee et al. 2020).

Although several mechanisms have been proposed, the pathogenesis of IFALD is not yet fully understood and warrants further investigation. Some of the most studied TPN-related mechanisms are those associated with the composition of lipid emulsions and subsequent lipotoxicity (Driscoll 2023). In this respect, the omega-6 to omega-3 fatty acids ratio and the amount of plant-derived phytosterols, especially high in soybean-based lipid emulsions, are of particular importance. While a high omega-6 to omega-3 fatty acid ratio promotes inflammation, a high abundance of phytosterols can affect bile acid metabolism (Zaloga 2015; Notz et al. 2022; Driscoll 2023). Consequently, intravenously administered lipids can cause both hepatic steatosis and cholestasis (Driscoll 2023). In addition, lipid overload can trigger severe saturated free fatty acids-mediated lipotoxicity, which is driven by mitochondrial oxidative stress, endoplasmic reticulum (ER) stress, activation of pro-inflammatory signaling cascades, and apoptosis (Cazanave and Gores 2010). Similarly, excessive glucose infusion may compromise mitochondrial fatty acid beta-oxidation and promote hepatic de novo lipogenesis, hence contributing to steatosis (Gabe and Culkin 2010; Kovacic and Somanathan 2013). Additional mechanisms involved in glucose toxicity are oxidative stress and inflammation (Kovacic and Somanathan 2013). Micronutrients (i.e*.,* manganese and copper) have been proposed to inflict liver damage secondary to mitochondrial dysfunction, oxidative stress, disruption in intracellular calcium and iron metabolism, inflammation, protein misfolding, ER stress, and apoptosis (Howard et al. 2007; Olson et al. 2019; Chandra and Keshavkant 2021; Pajarillo et al. 2022).

Thus far, IFALD has been mainly investigated in clinical patients and animal experimentation. Despite providing crucial insight into pathophysiology, clinical studies are often conducted in patients with underlying pathological conditions, which challenges the distinction between driving mechanisms and causes (Zafirovska et al. 2023; Tabone et al. 2024). TPN-related animal research has primarily focused on the use of surgical and preterm rodent and pig models, which has provided critical findings on pathophysiological processes underlying liver injury in the context of IFALD (Sangild et al. 2014; Burrin et al. 2020). Although useful, the translational value of such animal studies is questionable given well-known interspecies differences (Martić-Kehl et al. 2012). The shortcomings of clinical research and animal experimentation in the IFALD field could be overcome, at least in part, by the use of human-centered in vitro systems that allow in-depth investigation at the mechanistic level (Soldatow et al. 2013; Serras et al. 2021; Yang et al. 2023). In the present study, a three-dimensional (3D) spheroid co-culture system consisting of both human parenchymal and non-parenchymal liver cells was used to elucidate the mechanisms of TPN-associated liver injury.

## Materials and methods

### Chemicals and reagents

Unless stated otherwise, all chemicals and reagents were purchased from Sigma-Aldrich (St. Louis, MO, USA), prepared and used according to the manufacturer’s instructions. CELLSTAR^®^ culture 96-well ultra-low attachment (ULA) plates were obtained from Greiner Bio-One (Frickenhausen, Germany). Parenteral nutrition SmofKabiven^®^, trace elements concentrate Addaven^®^, liposoluble vitamins Vitalipid^®^ Novum Adult, and water-soluble vitamins Soluvit^®^ Novum, all produced by Fresenius Kabi (Kriens, Switzerland), were kindly provided by the Department of Clinical Nutrition of the Universitair Ziekenhuis Brussel (Brussels, Belgium).

### Cell culture and human liver spheroid co-culture characterization

**Fig. 1:**
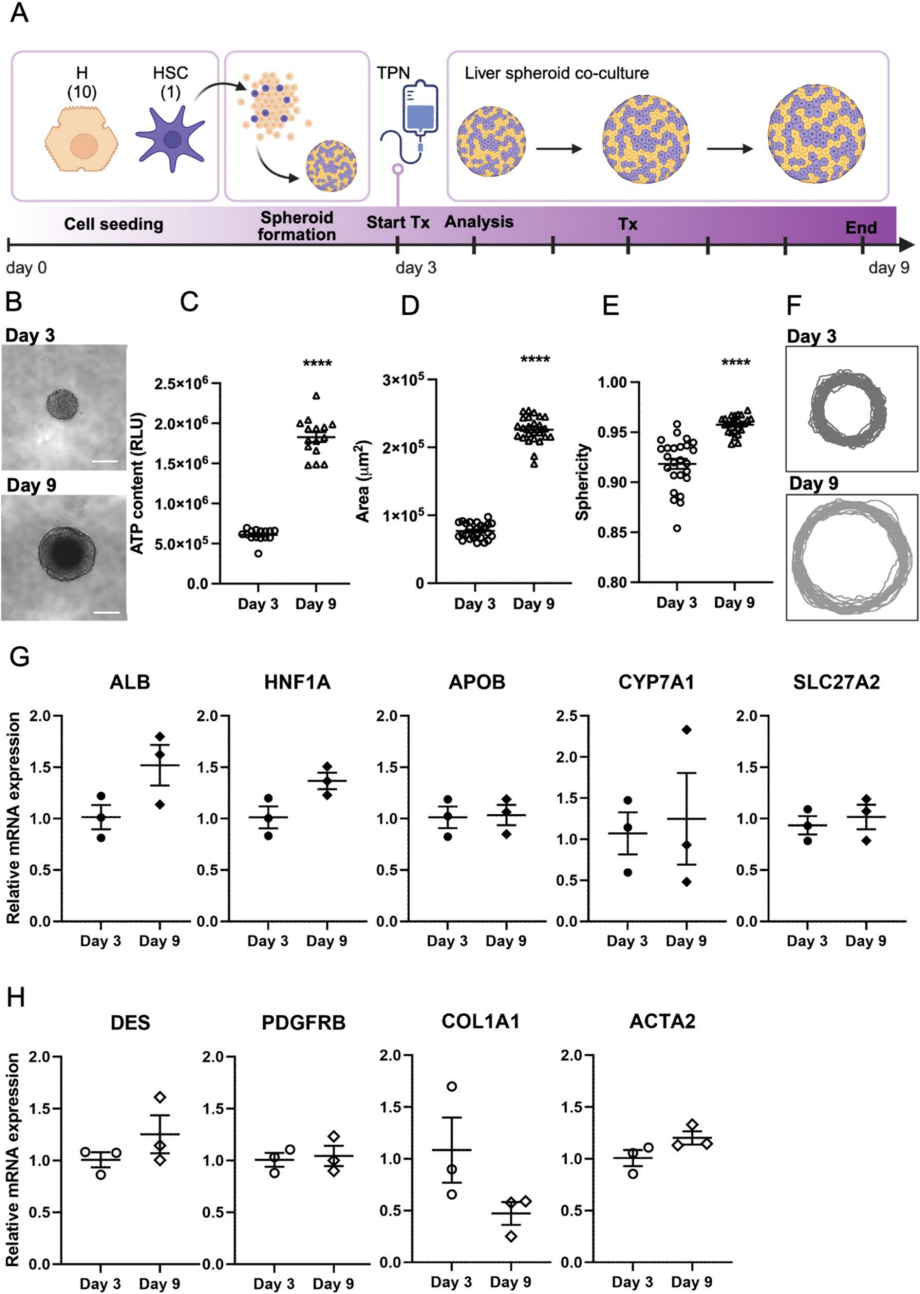
Generation and characterization of human liver spheroid co-cultures. **A** Schematic representation of the experimental setup. **B** Bright-field images of spheroids on day 3 (beginning of the treatment) and day 9 (end of the longest treatment); scale bar = 250 μm. **C** ATP content on day 3 and day 9. **D** Area of spheroids on day 3 and day 9. **E** Sphericity of spheroids on day 3 and day 9. **F** Overlapped contour outlining of spheroids edges on day 3 and day 9 of culture. **G** Relative mRNA expression levels of hepatocyte markers. **H** Relative mRNA expression levels of hepatic stellate cell markers. *H* hepatocyte, *HSC* hepatic stellate cell, *TPN* total parenteral nutrition, *Tx* treatment, *ALB* albumin, *HNF1A* hepatocyte nuclear factor-1 alpha, *APOB* apolipoprotein B, *CYP7A1* cytochrome P450 7A1, *SLC27A2* long-chain fatty acid transport protein 2, *DES* desmin, *PDGFRB* platelet-derived growth factor receptor beta, *COL1A1* alpha-1 type I collagen, *ACTA2* smooth muscle α-actin. Data are derived from three independent experiments and expressed as mean ± SEM. Statistical analysis was done using unpaired *t* test

Human liver spheroid co-cultures were set up using C3A cells, a clonal derivative of the human hepatoma HepG2 cell line (CRL-10741, ATCC, Manassas, VA, USA), and LX-2 cells, an immortalized activated human hepatic stellate cell (HSC) line (SCC064, Sigma-Aldrich). C3A and LX-2 cells were cultured in minimum essential medium (MEM) (Gibco, Waltham, MA, USA) supplemented with 1 mM sodium pyruvate, 1% non-essential amino acids (Gibco), 10% fetal bovine serum (Gibco), 100 U/ml penicillin, and 100 μg/ml streptomycin at 37 °C, at 5% (v/v) CO_2_. As schematically shown in Fig. 1A, liver spheroids were generated by seeding C3A and LX-2 cells at a ratio of 10:1 (500 C3A cells/well and 50 LX-2 cells/well in a total volume of 100 μL; day 0) on 96-well ULA plates for 72 h under gentle shaking, as described previously (Dos Santos et al. 2023). Spheroid growth over time was monitored by taking images using a Nikon Eclipse Ti-S microscope (Nikon, Tokyo, Japan) with a 10 × objective. Spheroid area, sphericity, and contours were determined by manually tracing the outlines of spheroids’ edges in ImageJ (version 1.54d; National Institutes of Health, Bethesda, MD, USA) from bright-field images taken from at least 25 spheroids on day 3 and day 9 following cell seeding. The spheroid area was extracted from the corresponding built-in measure. The spheroid sphericity was defined and calculated as the ratio of the circumference of a perfect circle with an area identical to that of the spheroid and the circumference of the spheroid itself. Spheroid contours were extracted as *XY*-coordinates in ImageJ and overlays of individual contours were prepared in R (version 4.2.0).

### Cell exposure to TPN and its single components

A sterile three-chamber bag of SmofKabiven^®^ parenteral nutrition (1497 mL), containing amino acid solution with electrolytes (750 mL), glucose (446 mL; 2.33 M), and lipid emulsion (281 mL), as well as trace elements concentrate Addaven^®^ (10 mL), and vitamins preparations Vitalipid^®^ Novum Adult and Soluvit^®^ Novum (10 mL) was prepared according to the manufacturer’s instructions and used for cell culture experiments. The TPN mixture prepared for in vitro testing reflects the quantity and ratios of all single components contained in a final mixture preparation used in clinical settings (Table 1). Starting from day 3 following cell seeding, human liver spheroid co-cultures were exposed to TPN and its components for a variable period, ranging from 24 to 144 h (Fig. 1A). MEM cell culture medium was used to prepare different concentrations of TPN and its single components.

**Table 1:** Concentrations of total parenteral nutrition and its components used for exposure of co-culture liver spheroids

Component	Abbreviation	Amount per bag in mL (%)	In vitro testing concentration range (%)
Total parenteral nutrition	TPN	1497 (100)	0.1–1–5–25–50
Amino acids and electrolytes	AAE	750 (50.1)	0.05–0.5–2.5–12.5–25
Glucose	G	446 (29.8)	0.03–0.3–1.5–7.5–15
Lipid emulsion	L	281 (18.8)	0.02–0.2–0.95–4.75–9.5
Addaven^®^ (trace elements)	TE	10 (0.67)	0.00067–0.0067–0.03–0.17–0.33
Vitalipid^®^ Novum Adult + Soluvit^®^ Novum (vitamins)	VIT	10 (0.67)	0.00067–0.0067–0.03–0.17–0.33

### Cell viability

Human liver spheroid co-cultures were exposed to TPN and its single components for 24, 48, 72, and 144 h. Cell viability was determined by measuring the total adenosine triphosphate (ATP) content using the CellTiter-Glo^®^ 3D Cell Viability Assay (G9682, Promega, Leiden, Netherlands) and by following manufacturer’s instructions. Briefly, 70 μL of cell culture medium per well was removed, 30 μL of Cell TiterGlo^®^ 3D reagent was added, and the spheroids were lysed by pipetting up and down vigorously. Following a 20-min incubation at 37 °C in the dark, 50 μL per well of the mixture lysate was transferred to a white plate (Lumitrac, Greiner Bio-One GmbH, Frickenhausen, Germany) and the bioluminescence signal was measured using SpectraMax iD3 Multi-Mode Reader (Molecular Devices, San Jose, CA, USA). At least 3 human liver spheroids per treatment condition and 15 for growth assessment over time were used to measure the total ATP.

### RNA extraction, cDNA synthesis, and real-time RT-qPCR analysis

Total RNA from approximately 65 human liver spheroids cultured for 3 days and 9 days (time points corresponding to the start of the treatments and the longest treatment duration, respectively) was isolated using the RNeasy Mini kit (Qiagen, Hilden, Germany) according to the manufacturer’s instructions and quantified using the NanoDrop^®^ ND-1000 spectrophotometer (ThermoFisher Scientific, Merelbeke, Belgium). The synthesis of complementary DNA (cDNA) was performed using 800 ng of total mRNA per sample and by means of the iScriptTM Reverse Transcription Supermix (Bio-Rad, Temse, Belgium). Real-time reverse transcription quantitative polymerase chain reaction (RT-qPCR) analysis was performed using 10 ng of cDNA, Taqman^®^ Fast Advanced Mastermix (Applied Biosystems, Waltham, MA, USA) and Taqman probes (*ALB*, Hs00609411_m1; *HNF1A*, Hs00167041_m1; *APOB*, Hs00181142_m1; *CYP7A1*, Hs00167982_m1; *SLC27A2*, Hs00167982_m1; *DES*, Hs00157258_m1; *PDGFRB*, Hs01019589_m1; *COL1A1*, Hs00164004_m1; *ACTA2*, Hs00426835_g1; *HPRT1*, Hs02800695_m1), following manufacturer’s instructions and using StepOnePlus™ real-time PCR system (Applied Biosystems, Waltham, MA, USA). Relative mRNA expression levels were calculated according to the Pfaffl method (Pfaffl 2001). *HPRT1* was used as a housekeeping gene for normalization purposes.

### RNA sequencing and transcriptome analysis

Total RNA from approximately 65 human liver spheroid co-cultures treated with 1% TPN for 24 and 144 h was extracted employing the RNeasy Mini kit (Qiagen), and only the RNA samples of high quality (RNA integrity number > 7.0) as measured using the 4150 TapeStation (Agilent Technologies, Santa Clara, CA, USA) were used. Library preparation and RNA sequencing were performed with BGI Genomics (Hong Kong, China) using DNBSEQ™ sequencing technology, as previously described (Drmanac et al. 2010). Sequence reads were checked for quality and filtered using SOAPnuke software (v1.5.2; BGI Genomics) (Cock et al. 2010; Chen et al. 2018b). The filtered clean reads were aligned to the reference human genome assembly GRCh38.p13 (version GCF_000001405.39_GRCh38.p13) using HISAT2 software (v2.0.4) and mapped to the reference transcripts using Bowtie2 software (v2.2.5), and the gene expression level of each sample was calculated using RSEM (v1.2.8) (Li and Dewey 2011; Langmead and Salzberg 2012). Differential gene expression (DEG) analysis was performed using the DESeq2 analysis method (Love et al. 2014). Transcripts with a log_2_-transformed gene expression fold change |log_2_(FC)|> 0.58 and adjusted *p* value (*Q* value) < 0.05 were deemed significantly differentially expressed. The Kyoto Encyclopedia of Genes and Genomes (KEGG) was used to perform the enrichment analysis of DEGs (Kanehisa 2000).

### Mitochondrial membrane potential detection

Liver spheroid co-cultures were exposed to TPN and its single components for 24 and 144 h. Mitochondrial membrane potential (MMP, ΔΨ_M_) was assessed using the cationic Rhodamine 123 probe (Ex/Em = 505/534 nm), accumulating in the negatively charged mitochondrial matrix, to stain liver spheroid co-cultures, as explained elsewhere (Dos Santos et al. 2023). The dye accumulation is inversely proportional to MMP. Briefly, after 24- or 144-h exposure to TPN and its single components, 8–12 human liver spheroid co-cultures were pooled, gently disaggregated into single-cell suspension using TrypLE™ Express (Gibco, Waltham, MA, USA), and, by pipetting up and down, washed with phosphate-buffered saline (PBS) and incubated with Rhodamine 123 (1.25 μM) for 30 min at 37 °C in the dark. Subsequently, cells were washed and resuspended in PBS and analyzed using Attune Acoustic Focusing Cytometer (Life Technologies, Carlsbad, CA, USA). Measured fluorescence was used to quantify relative MMP normalized against untreated control. As a positive control, human liver spheroid co-cultures exposed to carbonyl cyanide 4-(trifluoromethoxy) phenylhydrazone (FCCP) (20 μM) for 72 h were used (Sakamuru et al. 2016; Dos Santos et al. 2023).

### ER stress detection

Liver spheroid co-cultures were exposed to TPN and its single components for 24 and 144 h. ER stress was determined using thioflavin T (ThT; Ex/Em = 440/490 nm) to quantify intracellular protein aggregates (Verwilst et al. 2019; Dos Santos et al. 2023). Following exposure to TPN and its single components for 24 or 144 h, at least eight human liver spheroid co-cultures were pooled, gently disaggregated into single-cell suspension using TrypLE™ Express, and, by pipetting up and down, washed with PBS and stained with ThT (15 μM). Following 30 min of incubation at 37 °C in the dark, the suspension of single cells was washed once, resuspended in PBS and analyzed using the Attune Acoustic Focusing Cytometer. Detected fluorescence was used to quantify relative protein aggregates, and therefore ER stress, normalized against untreated control. Thapsigargin (Tg; 10 μM) treatment of human liver spheroid co-cultures for 72 h was used as a positive control (Lindner et al. 2020; Dos Santos et al. 2023).

### Intracellular reactive oxygen species detection

Intracellular reactive oxygen species (ROS) levels were measured using chloromethyl-2′,7′-dichlorofluorescein diacetate (CM-H_2_DCFDA; Invitrogen, Carlsbad, CA, USA), according to the manufacturer’s instructions. In short, after 2 h of exposure to TPN and its single components, human liver spheroid co-cultures were washed with PBS and stained with CM-H_2_DCFDA (10 μM). Following incubation of 25 min at 37 °C in the dark, human liver spheroid co-cultures were washed with PBS and lysed using 1% Triton X-100. A 100 μL of lysate per sample was transferred to a black 96 well-plate and the fluorescence signal was measured using SpectraMax iD3 Multi-Mode Reader (Ex/Em = 492/518 nm). Fluorescence values recorded were used to quantify relative intracellular ROS against untreated control. Human liver spheroid co-cultures exposed to menadione (100 μM) for 2 h were used as a positive control for ROS assessment (Criddle et al. 2006). At least six human liver spheroids per condition were used to measure the ROS levels.

### Apoptosis analysis

Liver spheroid co-cultures were exposed to TPN and its single components for 144 h. Apoptotic cells were detected using eBioscience™ Annexin V apoptosis detection kit (Invitrogen, Waltham, MA, USA) according to the manufacturer’s instructions. Following 144 h exposure to TPN, 12 human liver spheroid co-cultures per sample were pooled, washed with PBS, and gently disaggregated into single-cell suspension using TrypLE™ Express and by pipetting up and down. Subsequently, cells were washed with binding buffer and stained with FITC-conjugated annexin V. Following a 15-min incubation at room temperature in the dark, cells were washed with binding buffer, stained with propidium iodide (PI), and analyzed using Attune Acoustic Focusing Cytometer. Analyzed cells were expressed as a percentage of apoptotic cells (Annexin V^+^PI^−/+^). Human liver spheroid co-cultures exposed to camptothecin (CPT; 10 μM) for 48 h were used as a positive control for apoptosis assessment (Traganos et al. 1996; Dos Santos et al. 2023).

### Intracellular lipid accumulation detection using flow cytometry

Liver spheroid co-cultures were exposed to TPN and its single components for 24 and 144 h. Intracellular lipid accumulation in human liver spheroid co-cultures was quantified using BODIPY™ 493/503 neutral lipid dye and flow cytometry (Boeckmans et al. 2020). Briefly, following 24 or 144 h of exposure to TPN and its single components, at least eight human liver spheroid co-cultures were pooled, gently disaggregated into single-cell suspension using TrypLE™ Express, and, by pipetting up and down, washed with PBS and stained with BODIPY™ 493/503 neutral lipid dye (Thermo Fisher Scientific, Merelbeke, Belgium) (2 μM). Following 10 min of incubation at 37 °C in the dark, the suspension of single cells was analyzed using the Attune Acoustic Focusing Cytometer. Detected fluorescence was used to quantify intracellular lipid accumulation, normalized against untreated control. Spheroids treated with valproic acid sodium salt (VPA; 5 mM) for 48 h were used as a positive control (Bai et al. 2017).

### Fluorescent staining of intracellular lipids

Liver spheroid co-cultures were exposed to either TPN (5%) or lipid emulsion (0.95%) for 144 h and VPA (5 mM) for 48 h. To stain intracellular lipids, at least six human liver spheroid co-cultures per sample were pooled and washed briefly with PBS. Subsequently, human liver spheroid co-cultures were fixed with 4% (w/v) paraformaldehyde for at least 30 min at room temperature and thoroughly washed with PBS for 5 min. Next, spheroids were quickly embedded into a PolyFreeze tissue freezing medium and stored for 2 h at − 20 °C. Spheroid-containing solid frozen blocks were then sliced using a cryotome (MICROM GmbH HM 525, Walldorf, Germany). The obtained sections of 16 μm thickness were placed onto a microscope slide (SuperFrost^®^Plus, VWR, Leuven, Belgium) and immediately used for fluorescent staining. Following a short washing with PBS (5 min), sections were incubated with 2 μM BODIPY™ 493/503 neutral lipid dye (Thermo Fisher Scientific, Merelbeke, Belgium) for 15 min at room temperature in the dark, washed with PBS for 5 min, and imaged using a Nikon Eclipse Ti-S microscope with a 10 × objective.

### Data analysis

All data are presented as mean ± standard error of the mean (SEM). Statistical analysis was performed using one-way analysis of variance (ANOVA) followed by Dunnett’s multiple comparison test or an unpaired *t* test. A *p* value < 0.05 was considered significant. GraphPad Prism (version 10.1.1; GraphPad Software Inc., La Jolla, CA, USA) was used for statistical analysis. All experiments were repeated independently at least three times.

## Results

### Human liver spheroid co-culture establishment and characterization

Human liver spheroid co-cultures were successfully generated using 500 C3A cells and 50 LX-2 cells in a ratio of 10 to 1 cultured on ULA plates for 72 h (3 days). The average diameter of each spheroid after 3 days of culture, which corresponds to the starting point for TPN treatment, was approximately 200 μm, while the size of the growing spheroids reached approximately 500 μm in diameter after 9 days of culture, corresponding to the longest TPN exposure time of 144 h (Fig. 1A, B). ATP content and spheroid area measured on day 3 and day 9 of the culture period showed growth over time (Fig. 1C, D) with sustained sphericity (Fig. 1E, F). Moreover, the expression of typical markers of hepatocytes (Fig. 1G) and HSCs (Fig. 1H) remained stable over time suggesting that both cell types preserve their function in the 3D spheroid configuration. The expression levels of the housekeeping gene HPRT1 used for normalization were stable and did not change over time (Fig. S1).

### Effect of TPN exposure on cell viability

**Fig. 2:**
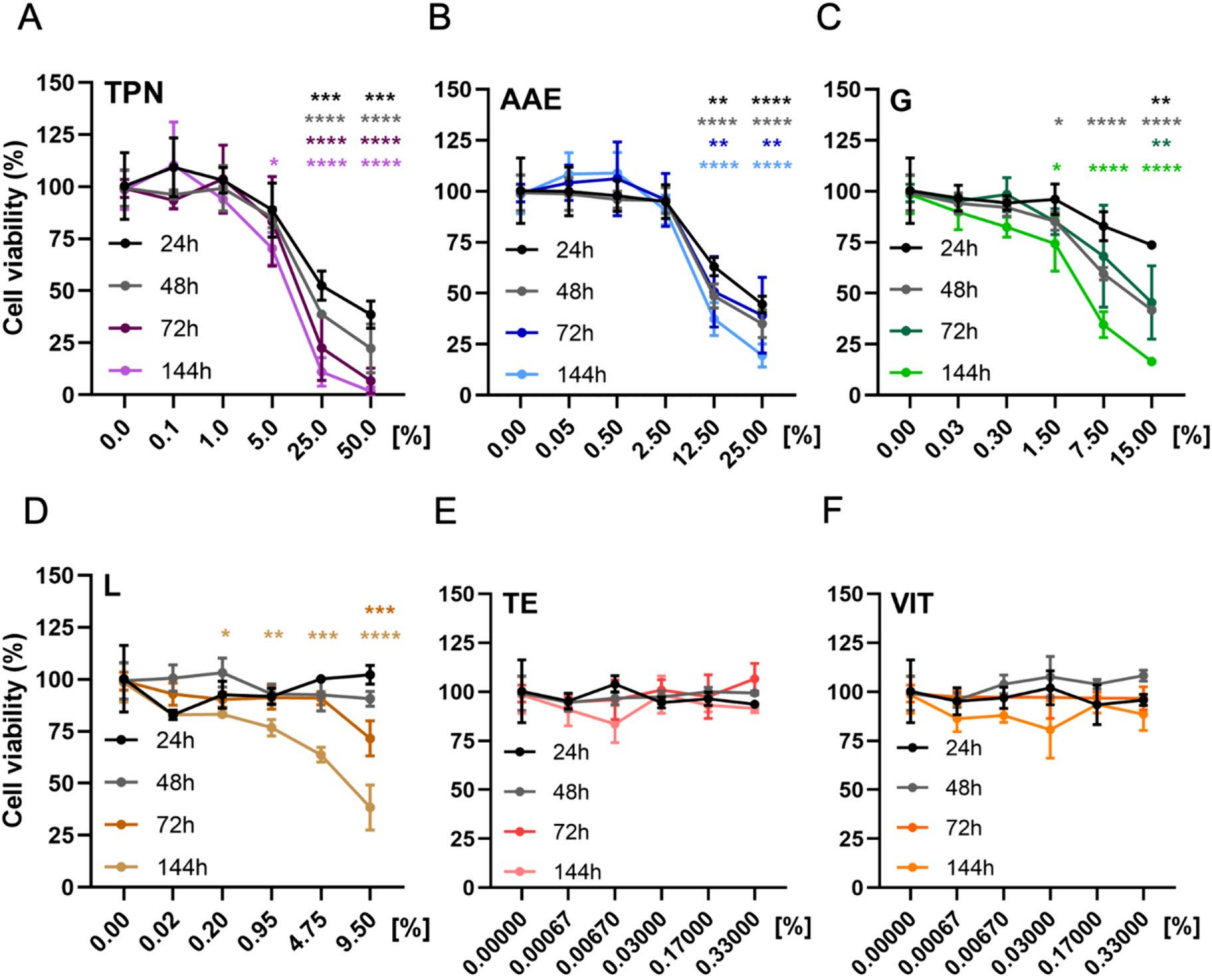
The effect of TPN and its main components on cell viability of human liver spheroid co-cultures. The effect of **A** total parenteral nutrition, **B** amino acids and electrolytes, **C** glucose, **D** lipid emulsion, **E** trace elements, and **F** vitamins, after 24, 48, 72, and 144 h of exposure. *TPN* total parenteral nutrition, *AAE* amino acids and electrolytes, *G* glucose, *L* lipid emulsion, *TE* trace elements, *VIT* vitamins. Data are derived from three independent experiments performed in triplicate and expressed as mean ± SEM. *p < 0.05, **p < 0.01, ***p < 0.001, ****p < 0.0001 (1-way ANOVA followed by Dunnett’s multiple comparison test, using untreated cells at a corresponding exposure time as a control)

To assess the effect of TPN on cell viability, the ATP assay was performed following different exposure times ranging from 24 to 144 h. Concentrations of up to 1% of TPN did not affect cell viability regardless of the exposure time (Fig. 2A). A concentration of 5% caused a reduction in cell viability of around 25% only after 144 h of treatment. Concentrations of 25% and 50% appeared to be highly toxic, since cell viability was reduced by 50%-100% in a time- and concentration-dependent manner. The solution of amino acids and electrolytes also appeared to have no effect up to 2.5% at all exposure times tested, while concentrations of 12.5% and 25% caused a time-dependent reduction of cell viability by 40%-80% (Fig. 2B). Exposure to increasing concentrations of glucose confirmed that the two highest concentrations (7.5% and 15%) were cytotoxic, especially after 72 and 144 h of treatment, with a drop in cell viability by 30%–85% (Fig. 2C). A reduction of cell viability of approximately 25% was observed for 1.5% glucose after 144 h of exposure. Lipids appear to have no effect for short-term exposures of 24–48 h (Fig. 2D). At higher exposure times (144 h), there was a reduction of cell viability starting from a 15% reduction at the concentration of 0.2% of lipids, and reaching a 60% reduction of cell viability at lipids concentration of 9.5%. Both trace elements (Fig. 2E) and vitamins (Fig. 2F) did not significantly affect cell viability at any of the concentrations and exposure times tested.

### Effect of short-term and long-term TPN exposure on the liver spheroid transcriptome

**Fig. 3:**
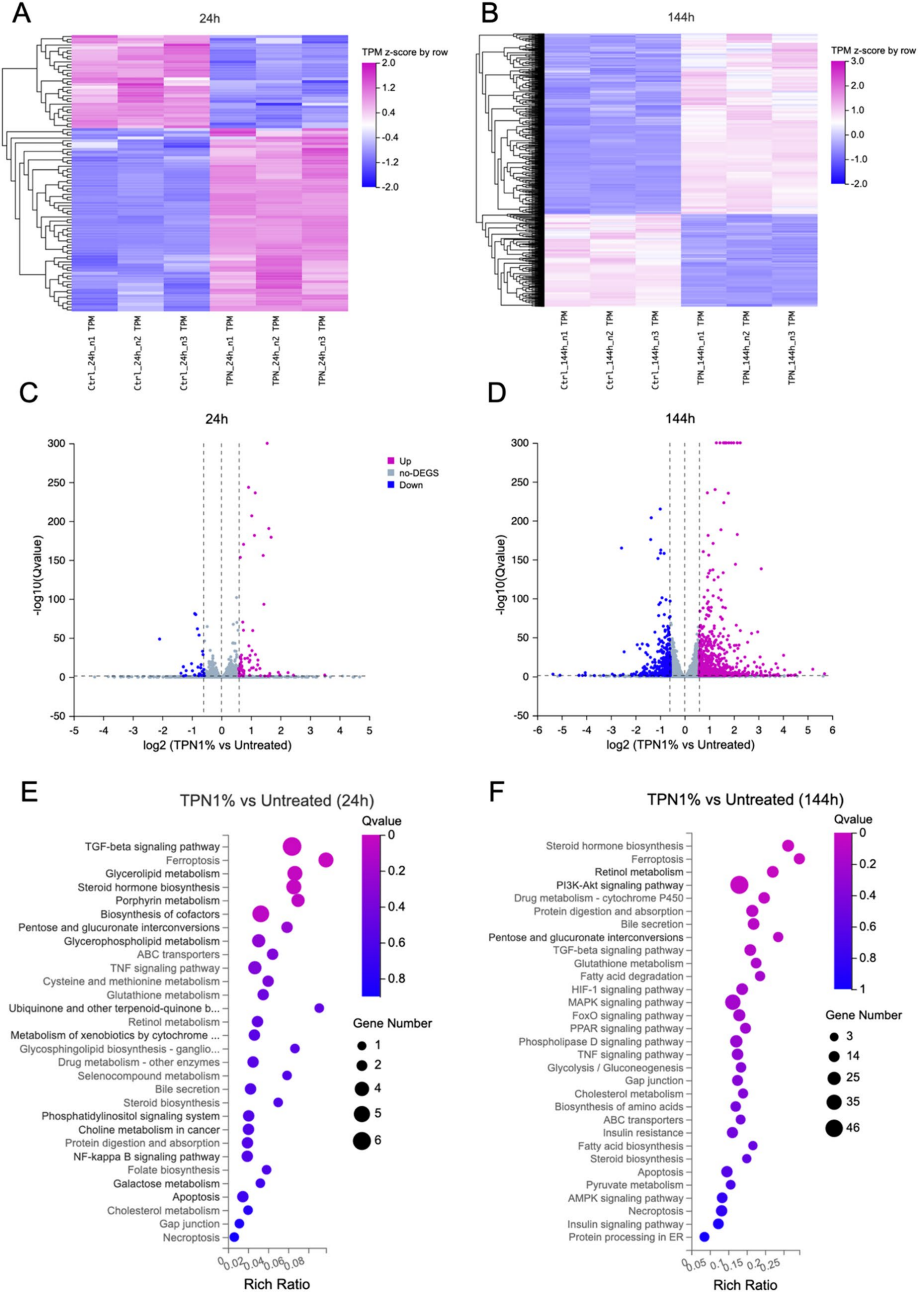
Transcriptomic profile of human liver spheroid co-cultures exposed to TPN. *Z*-score hierarchical clustering heat map visualization of differentially expressed genes (DEGs) after **A** 24 h of exposure and **B** 144 h of exposure to 1% TPN. Volcano plots illustrating DEGs after **C** 24 h of exposure and **D** 144 h of exposure to 1% TPN. Significantly downregulated genes are indicated in blue, and significantly upregulated genes in purple. The −log_10_ (*Q* value) is plotted against the log_2_ (fold change TPN 1%/untreated control). Only significant DEGs (|log_2_(FC)|> 0.58 and *Q* value < 0.05) are reported. Kyoto Encyclopedia of Genes and Genomes (KEGG) enrichment analysis illustrating the most affected metabolic and molecular signaling pathways after **E** 24 h of exposure and **F** 144 h of exposure to 1% TPN. The horizontal axis represents the rich factor (number of DEGs in a pathway/number of all annotated genes in the given pathway), while the vertical axis represents the enriched pathway name. The color scale indicates different thresholds of the *Q* value, and the size of the dot indicates the number of genes corresponding to each pathway. Data are derived from three independent experiments

RNA sequencing and transcriptomics analysis of human liver spheroid co-cultures exposed to 1% TPN for 24 and 144 h were performed to gain a deeper insight into molecular signatures and pathways most affected after short-term and long-term exposure to TPN. A concentration of 1% was selected based on the cell viability results and preliminary data of functionality assays, which suggested that at the concentration of 1% the effects of TPN could be detected, without compromising cell viability. A heat map representation of DEGs showed that both untreated control samples and the TPN-exposed samples display similar expression patterns among each other both for 24- and 144-h exposure times (Fig. 3A, B). The numbers of DEGs were higher after 144 h of exposure compared to 24 h of exposure to TPN, indicating that the duration of exposure affects gene expression (Fig. 2C, D). Transcriptomic profiling revealed that 98 and 1,300 genes were differentially expressed following 24 and 144 h of treatment, respectively. DEGs at both time points were subjected to KEGG enrichment analysis in order to determine the most affected molecular pathways and biological processes (Kanehisa 2000). Various metabolic and signaling pathways seemed to be affected, especially after 144 h of exposure (Fig. 3E, F). Among those were several pathways involved in metabolic processes (i.e., fatty acids and cholesterol metabolism, steroid biosynthesis, glycolysis, gluconeogenesis, insulin signaling, amino acids and protein metabolism, glutathione metabolism, and PPAR signaling), cellular responses to stress (i.e., PI3K-Akt, MAPK, HIF-1, TGF-β and FoxO), and cell death processes (i.e*.,* apoptosis, necroptosis, ferroptosis, and TNF-α signaling) (Figure S2-10). The altered expression of several nuclear receptors, transcription factors, and receptors involved in peroxisomal fatty acid beta-oxidation, indicative of steatosis development, is depicted in Fig. S11.

### Cellular stress response to TPN exposure

**Fig. 4:**
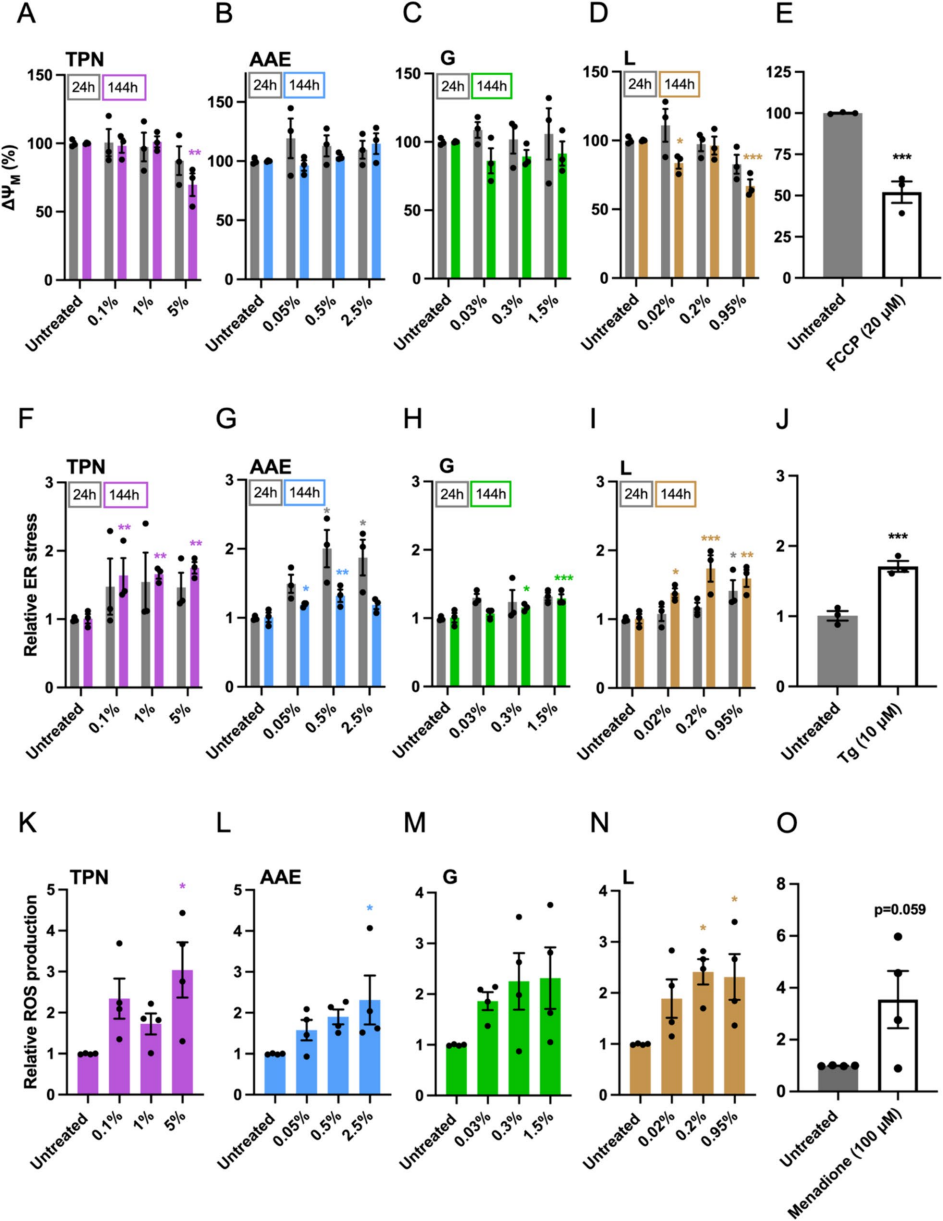
Effect of TPN and its components on mitochondrial dysfunction, endoplasmic reticulum stress, and oxidative stress. Mitochondrial membrane potential (ΔΨ_M_) after 24 and 144 h of exposure to increasing concentrations of **A** TPN, **B** amino acids and electrolytes, **C** glucose, and **D** lipid emulsion. **E** Mitochondrial membrane potential (ΔΨ_M_) after exposure to 20 μM carbonyl cyanide 4-(trifluoromethoxy) phenylhydrazone for 72 h. Intracellular protein aggregates indicative of endoplasmic reticulum stress after 24 and 144 h of exposure to increasing concentrations of **F** TPN, **G** amino acids and electrolytes, **H** glucose, and **I** lipid emulsion. **J** Endoplasmic reticulum stress after exposure to 10 μM thapsigargin for 72 h. Intracellular reactive oxygen species production after 2 h of exposure to increasing concentrations of **K** TPN, **L** amino acids and electrolytes, **M** glucose, and **N** lipid emulsion. **O** Intracellular reactive oxygen species generation after exposure to 100 μM menadione for 2 h. *TPN* total parenteral nutrition, *AAE* amino acids and electrolytes, *G* glucose, *L* lipid emulsion, *FCCP* carbonyl cyanide 4-(trifluoromethoxy) phenylhydrazone, *ROS* reactive oxygen species, *Tg* thapsigargin. Data are derived from at least three independent experiments expressed as mean ± SEM. *p < 0.05, **p < 0.01, ***p < 0.001 (1-way ANOVA followed by Dunnett’s multiple comparison test using untreated cells at a corresponding exposure time as a control; unpaired *t* test was used for positive control treatments)

MMP or ΔΨ_M_ was measured following exposures of human liver spheroid co-cultures to TPN and its components. Only the highest concentration of TPN (5%) and lipids (0.95%) tested for 144 h of exposure induced a reduction of the MMP by 30% and 33%, respectively (Fig. 4A, D). Glucose and amino acids and electrolytes did not affect MMP at any concentration or time point tested (Fig. 4B, C). Positive control FCCP induced approximately a 50% reduction of MMP (Fig. 4E). Next, intracellular protein aggregates were measured to determine ER stress. It was found that after 144 h of exposure, all TPN concentrations tested increased ER stress response by 65%–75% (Fig. 4F). Amino acids and electrolytes (0.5%), as well as glucose (1.5%), caused not more than a 30% increase in ER stress on 144 h of exposure (Fig. 4G, H). Lipid emulsion, on the other hand, caused an increase in ER stress after 144 h of exposure by 35%-70% for all concentrations, and even a 40% increase for the concentration of 0.95% after 24 h of exposure (Fig. 4I). A 70% increase in ER stress was also observed for positive control Tg (Fig. 4J). Intracellular ROS were measured as an indicator of oxidative stress following a short exposure time of 2 h. The data suggest that there is a trend in TPN causing ROS generation, especially at the concentration of 5% for which a threefold increase was detected (Fig. 4K). Similar results were found for amino acids and electrolytes and glucose with a clear trend toward increased oxidative stress with a 2.3-fold increase for the highest concentrations (Fig. 4L, M). A 2.2-fold increase in ROS levels was also detected for lipid emulsion (0.2% and 0.95%) (Fig. 4N), as well as a steep 3.5-fold increase for positive control menadione (Fig. 4O).

### Cell death response to TPN exposure

**Fig. 5:**
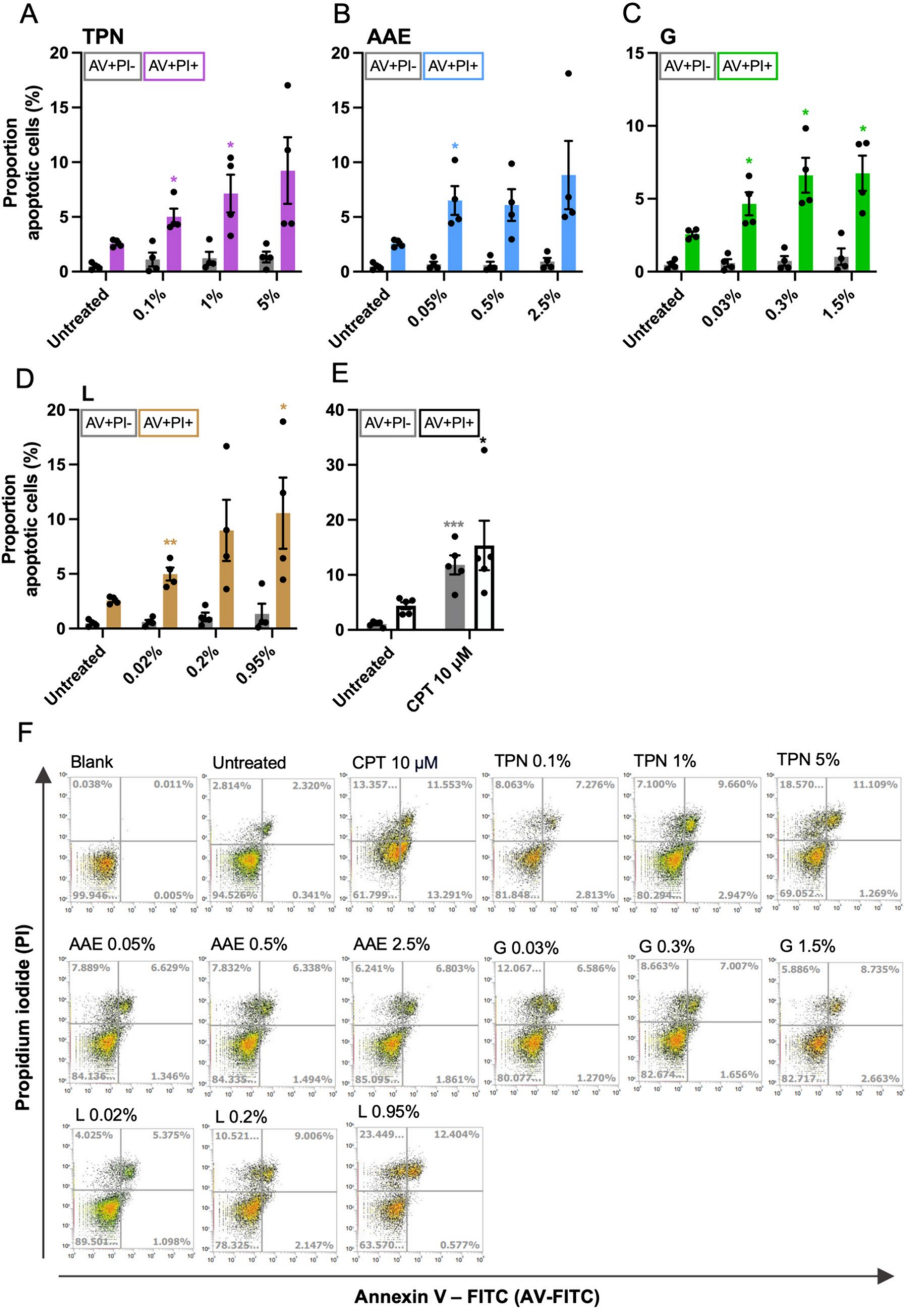
Effect of TPN and its components on apoptosis. Proportion (%) of early (AV^+^PI^−^) and late (AV^+^PI^+^) apoptotic cells after 144 h of exposure to **A** TPN, **B** amino acids and electrolytes, **C** glucose, and **D** lipid emulsion. **E** Proportion (%) of early (AV^+^PI^−^) and late (AV^+^PI^+^) apoptotic cells after exposure to 10 μM camptothecin for 48 h. **F** Representative dot plots of Annexin V and propidium iodide staining indicating proportion (%) of early (AV^+^PI^−^) and late (AV^+^PI^+^) apoptotic cells after 144 h exposure to total parenteral nutrition, amino acids and electrolytes, glucose, and lipid emulsion, and after exposure to 10 μM camptothecin for 48 h. *AV* Annexin V, *PI* propidium iodide, *TPN* total parenteral nutrition, *AAE* amino acids and electrolytes, *G* glucose, *L* lipid emulsion, *CPT* camptothecin. Data are derived from four independent experiments and expressed as mean ± SEM. *p < 0.05, **p < 0.01, ***p < 0.001 (1-way ANOVA followed by Dunnett’s multiple comparison test using corresponding early or late apoptotic cells in untreated conditions as a control)

Induction of apoptosis was measured using Annexin V and PI combined staining to distinguish between early and late apoptotic cells following exposure to TPN and components for 144 h. While only a minor increase in early apoptotic cells was observed for all treatment conditions, a significant increase in late apoptotic cells (Annexin V^+^PI^+^) was detected for several concentrations tested. A two to fourfold concentration-dependent increase for TPN and lipid emulsion (Fig. 5A, D, F), a 3.5-fold increase for amino acids and electrolytes (Fig. 5B, F), and a 1.8- to 2.6-fold increase for glucose (Fig. 5C, F) was observed. The exposure to CPT, used as a positive control, induced a tenfold increase in early apoptotic cells and a 3.5-fold increase in the number of late apoptotic cells (Fig. 5E, F).

### Steatotic response to TPN exposure

**Fig. 6:**
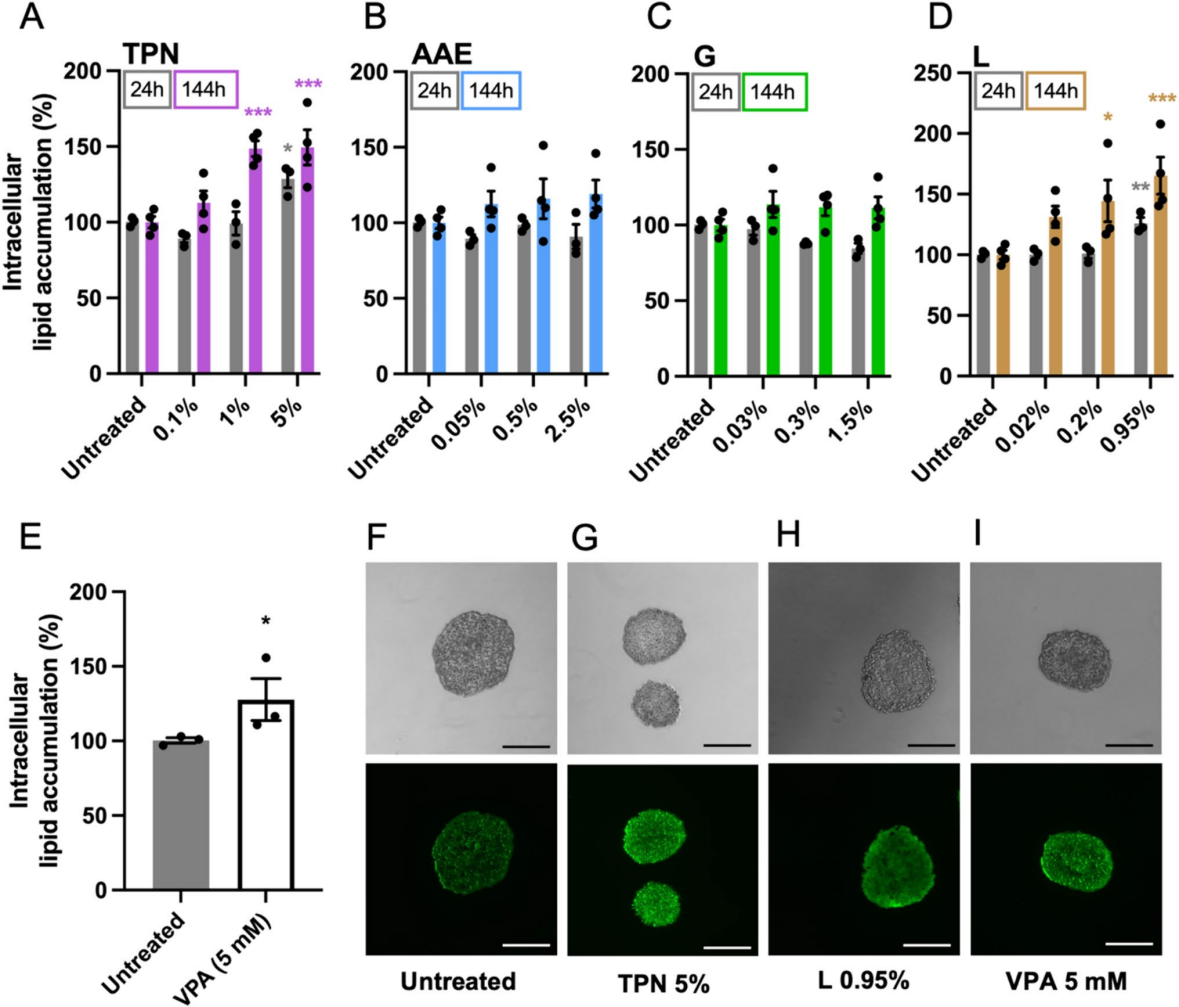
Effect of TPN and its components on steatosis. Quantification of intracellular lipid accumulation after 24 and 144 h of exposure to increasing concentrations of **A** TPN, **B** amino acids and electrolytes, **C** glucose, and **D** lipid emulsion. **E** Quantification of intracellular lipid accumulation after exposure to 5 mM valproic acid for 48 h. Representative fluorescence images of intracellular lipid staining with BODIPY™ 493/503 neutral lipid dye of **F** untreated spheroids, after 144 h of exposure to **G** 5% of TPN, **H** 0.95% of lipid emulsion, and **I** after exposure to valproic acid for 48 h; scale bar = 250 μm. *TPN* total parenteral nutrition, *AAE* amino acids and electrolytes, *G* glucose, *L* lipid emulsion, *VPA* valproic acid. Data are derived from at least three independent experiments and expressed as mean ± SEM. *p < 0.05, **p < 0.01, ***p < 0.001 (1-way ANOVA followed by Dunnett’s multiple comparison test using untreated cells at a corresponding exposure time as a control; unpaired *t* test was used for positive control treatment with valproic acid)

To assess the induction of steatosis, intracellular lipid accumulation was measured both after 24 and 144 h of exposure to TPN. A 30% increase in intracellular lipid accumulation was observed for 24-h exposure to 5% TPN, and a 50% increase for 1% and 5% TPN after 144 h of exposure (Fig. 6A), suggesting that the TPN-induced steatosis is a time-dependent rather than a dose-dependent effect. Amino acids and electrolytes (Fig. 6B) and glucose (Fig. 6C) did not cause any significant increase in steatosis regardless of the exposure time. However, lipid emulsion, similarly to TPN, induced an increase of 25% for the highest tested concentration after 24 h of treatment, and a 45% and a 65% increase in steatosis for 0.2% and 0.95% concentrations of lipid emulsion, respectively, for 144 h of exposure (Fig. 6D). A 30% increase in intracellular lipid accumulation was also detected after exposure to VPA, a well-known steatogenic drug (Fig. 6E). Fluorescent staining of intracellular neutral lipids confirmed that steatosis was induced by TPN, lipid emulsion, and VPA (Fig. 6F–I).

## Discussion

TPN is a life-saving nutritional support effective in counteracting malnutrition and assisting patients to reach and maintain a proper nutritional status (Berlana 2022). Notwithstanding its major benefits, the number and severity of TPN-associated complications cannot be disregarded. IFALD is one of the most common TPN-associated metabolic complications. The pathogenesis of IFALD is complex and underlying mechanisms remain elusive (Sobotka and Camilo 2009; Berlana 2022; Mihajlovic et al. 2024). Most of the known mechanisms described to contribute to IFALD are linked to toxicity of single components or inadequate administration of TPN (Berlana 2022). Among those, lipotoxicity is one of the most documented observations related to the composition of lipid emulsions (i.e.,, phytosterol content, omega-6 fatty acid content), which contribute to liver injury (Driscoll 2023). Other IFALD mechanisms are those linked to glucose toxicity as well as injury mediated by micronutrient toxicity (e.g., manganese, copper, aluminum) or nutrient deficiency (e.g*.,* carnitine, choline, vitamin E) (Howard et al. 2007; Gabe and Culkin 2010; Berlana 2022; Zafirovska et al. 2023). Liver damage also seems to be dictated by the patient’s health status including lack of enteral feeding, gut dysbiosis, short bowel syndrome, central line infections, and immature bile acid metabolism and enterohepatic circulation occurring in preterm neonates (Di Dato et al. 2022; Rosseel et al. 2023; Zafirovska et al. 2023).

**Fig. 7:**
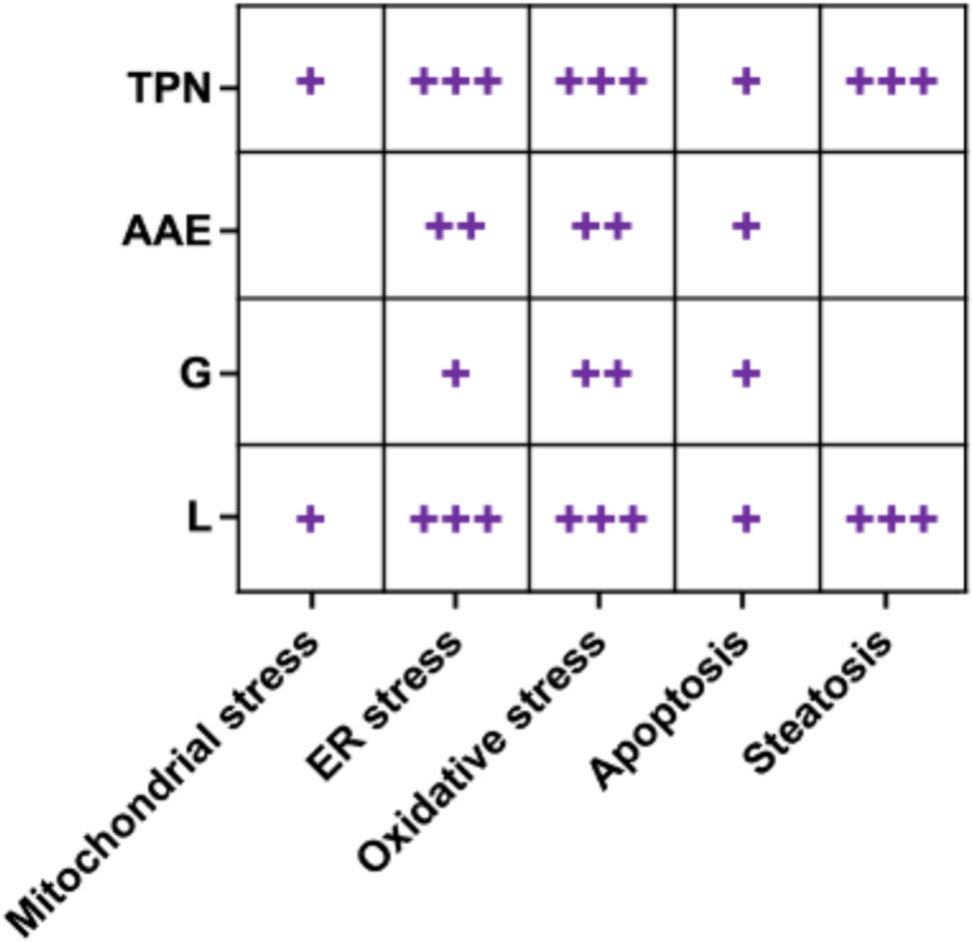
Qualitative summary of the results regarding the effects of TPN and its single components on the induction of mitochondrial stress, endoplasmic reticulum stress, oxidative stress, apoptosis, and steatosis. The positive impact of each component on a certain biological event assessed is indicated with a symbol + (i.e., the higher the number of + , the higher the impact on a specific event). No impact is indicated with a blank spot. *TPN* total parenteral nutrition, *AAE* amino acids and electrolytes, *G* glucose, *L* lipid emulsion, *ER stress* endoplasmic reticulum stress

In the present study, human liver spheroid co-cultures were used to study the mechanisms involved in IFALD. The results suggested that lipotoxicity plays a major role in TPN-associated liver damage. Detected intracellular lipid accumulation and subsequent cellular damage are consistent with observations of steatosis secondary to lipid overload and high infusion rates that exceed the hepatic metabolization rate of fatty acids and phospholipids (Meisel et al. 2011; Moon et al. 2017; Jordan et al. 2020). Identified lipotoxicity-mediating mechanisms include ER stress, mitochondrial dysfunction, oxidative stress, and apoptosis, complying with previous reports describing fatty acids- and/or triglyceride-induced hepatic injury (Wei et al. 2006; Cazanave and Gores 2010; Cao et al. 2012; Lee et al. 2015; Ress 2016; Liu et al. 2018; Maitiabula et al. 2022; Fromenty and Roden 2023; Zheng et al. 2023). Although it has been reported that infusion of higher amounts of glucose and amino acids can also bear steatotic potential, no direct effect of either glucose or amino acids on intracellular lipid accumulation was observed in the present study (Villanueva-Ortega et al. 2020; Gunnar et al. 2023). Nonetheless, the results of this study support available scientific data regarding mechanisms of glucose-mediated and amino acids-mediated toxicity, specifically those involved in oxidative stress, apoptosis and alterations of liver metabolic processes (Kawahito et al. 2009; Lynch and Adams 2014; Zhenyukh et al. 2017; Martínez et al. 2017; Panahi et al. 2018; Acito et al. 2020; Liu et al. 2023). High glucose levels have also been suggested to amplify fatty acid-induced ER stress and to contribute synergistically to lipotoxicity (Bachar et al. 2009; Yin et al. 2015). A qualitative summary of the results of this study performed for comparative purposes indicates that the amino acids and electrolytes solution appears to be the least toxic among all components, only slightly inducing oxidative stress and ER stress (Fig. 7). Similarly, glucose seems rather innocuous, exerting only minor adverse effects associated with oxidative stress and mitochondrial dysfunction. On the other hand, lipid emulsion is the component that majorly affects almost all parameters tested in the present study, in particular induction of ER stress and steatosis, and to a certain degree apoptosis, thereby significantly contributing to the overall adverse outcome of TPN on liver cells.

Key events evaluated in the present study and shown to be involved in TPN-associated liver damage, as well as results of transcriptomic analysis, are consistent with hepatotoxicity-related adverse outcome pathways (AOP), especially AOP of liver steatosis (Vinken 2013; Ankley and Edwards 2018; Knapen et al. 2018; Arnesdotter et al. 2021). Several molecular initiating events in the AOP network for liver steatosis, including modulation of nuclear receptors (i.e., aryl hydrocarbon receptor, liver X receptor, pregnane X receptor), suppression of transcription factors (i.e., hepatocyte nuclear factor 4 alpha, nuclear factor erythroid 2-related factor 2), and inhibition of peroxisomal fatty acid beta-oxidation (i.e., decreased activation of PPARs), also emerged in the transcriptomics analysis of the present study, further supporting the notion of TPN-mediated liver injury (Mellor et al. 2016; Arnesdotter et al. 2021). These molecular initiating events are known to affect lipid metabolism at different levels, and the RNA-sequencing data confirmed that various pathways, such as fatty acid degradation and biosynthesis, cholesterol metabolism, PPAR signaling and the increased hepatic influx of fatty acids were affected by TPN, indicating steatosis development (Mellor et al. 2016). Similar to previous reports, the results of the current study support the findings that perturbations in metabolic pathways are among the key effects of TPN exposure. In particular, the pathways related to insulin signaling, AMPK or FoxO are known to regulate metabolic processes, such as glycogenesis, gluconeogenesis, de novo lipogenesis and fatty acid beta-oxidation, thus contributing to steatotic injury (Mihaylova and Shaw 2011; Guthrie 2022; Jiang et al. 2022). Furthermore, the transcriptomic analysis showed that different types of programmed cell death were enriched in TPN-treated cells, including apoptosis, necroptosis and ferroptosis. The latter displayed a greater degree of pathway enrichment among the induced cell death pathways, which is in accordance with emerging studies demonstrating an important role for ferroptosis in IFALD pathogenesis (Jiang et al. 2022; Cheng et al. 2022).

Indicative of TPN-induced hepatic injury is the observation of significant enrichment of the TGF-β signaling pathway. Profibrogenic TGF-β is known to be orchestral in inducing activation and transdifferentiation of HSCs and extracellular matrix production, being hallmarks of liver fibrosis (Dewidar et al. 2019). An additional notion supporting the role of HSCs in TPN-induced liver injury is the enrichment of the pathway of retinol metabolism and extracellular matrix-receptor interactions (Carmona et al. 2019; Dewidar et al. 2019). These results underscore the potential impact of HSCs in contributing to TPN-induced liver injury and warrant further investigation into their role in the molecular pathogenesis of IFALD.

Despite providing important molecular and cellular insights into mechanisms of liver injury secondary to TPN exposure, a limitation of the present study is the lack of direct clinical translation. This is particularly reflected in attempts to correlate the dosages of TPN used in vitro to those observed in clinics during a TPN regimen. Computational toxicology methods that predict chemical toxicity and correlate in vitro and in vivo concentrations are available and could aid in the efforts needed to increase clinical relevance (Rodríguez-Belenguer et al. 2023a, b). In particular, physiologically based pharmacokinetic (PBPK) modeling can facilitate quantitative in vitro to in vivo extrapolation (QIVIVE) and allow to combine in silico and in vitro parameters and convert in vitro concentration–response curves into relevant in vivo exposures (Rietjens et al. 2011; Wilk-Zasadna et al. 2015; Chen et al. 2018a). However, the PBPK modeling of complex mixtures, such as TPN, containing a myriad of metabolically active substances and nutrients with different physicochemical and pharmacokinetic properties, remains a challenge and requires significant efforts, availability of appropriate modeling platforms, and interdisciplinary collaborations to perform accurate and clinically relevant modeling (Groothuis et al. 2015; Wilk-Zasadna et al. 2015; Paini et al. 2019; Moreau et al. 2022).

In conclusion, the present study confirmed the involvement of several key events in TPN-induced liver damage and indicated that lipid emulsion, among all major TPN components, seemed to be the main culprit of the observed adverse outcomes. A combination of advanced heterotypic cell models, suitable in vitro and omics analysis, systems toxicology approaches, including AOPs and their networks and PBPK modeling holds great promise for advancing the translational research oriented toward unraveling further IFALD mechanisms and encouraging the safe use of TPN.

### Supplementary Information

Below is the link to the electronic supplementary material.

Supplementary file1 (DOCX 4624 kb)

## Data Availability

The RNA-seq datasets generated during the current study are available in the Gene Expression Omnibus (GEO) repository, https://www.ncbi.nlm.nih.gov/geo/query/acc.cgi?acc=GSE264357. All other data generated or analysed during this study are included in this published article and its supplementary information files.
