# Supplementary material for: Investigation of parenteral nutrition-induced hepatotoxicity using human liver spheroid co-cultures
Source: Arch Toxicol. 2024 May 14;98(9):3109–26. doi: 10.1007/s00204-024-03773-8 (PMC11324701; doi:10.1007/s00204-024-03773-8)
Supplement: Supplementary file 1 — Supplementary file1 (DOCX 4624 kb) [file 204_2024_3773_MOESM1_ESM.docx]

**Investigation of parenteral nutrition-induced hepatotoxicity using human liver spheroid co-cultures**

Milos Mihajlovic^1^, Sybren De Boever^1^, Andrés Tabernilla^1^, Ellen Callewaert^1^, Julen Sanz-Serrano^1^, Anouk Verhoeven^1^, Amy Maerten^1^, Zenzi Rosseel^2,3^, Elisabeth De Waele^3,4^, Mathieu Vinken^1^

^1^ Department of Pharmaceutical and Pharmacological Sciences, Vrije Universiteit Brussel, Brussels, Belgium

^2^ Department of Pharmacy, Universitair Ziekenhuis Brussel (UZ Brussel), Brussels, Belgium

^3^ Department of Clinical Nutrition, Universitair Ziekenhuis Brussel (UZ Brussel), Brussels, Belgium

^4^ Faculty of Medicine and Pharmacy, Vrije Universiteit Brussel, Brussels, Belgium

**Supplementary Material**


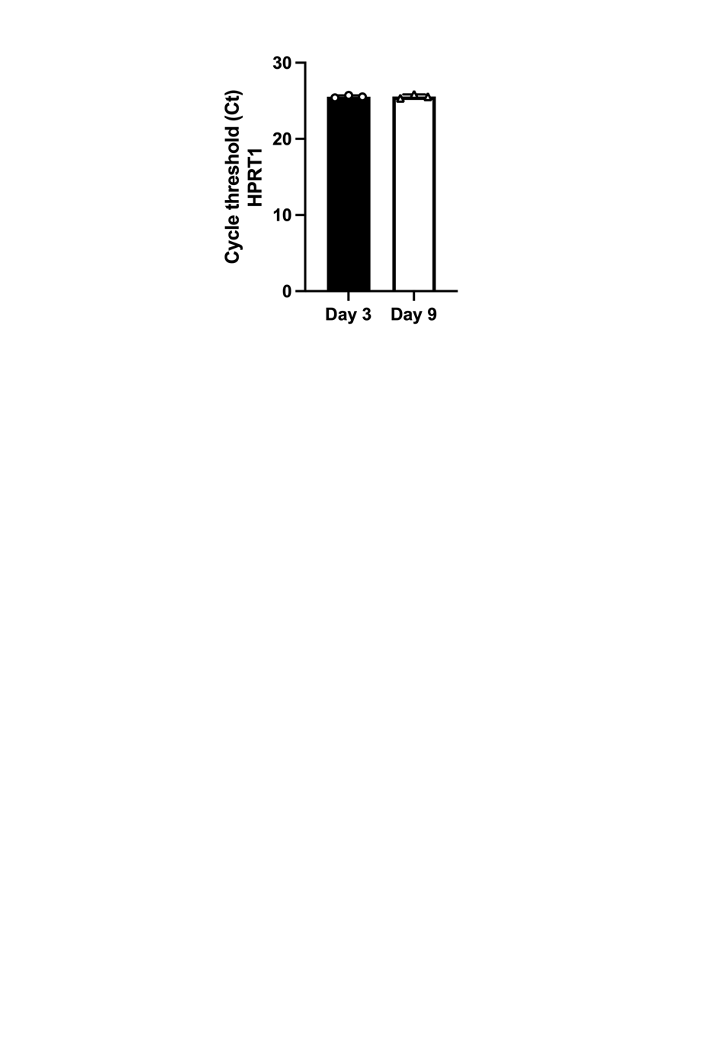


**Figure S1.** Cycle threshold (Ct) values for HPRT1 on day 3 and day 9 of spheroid cell culture. HPRT1 expression was stable, with no significant differences between the 2 time points. Data are derived from 3 independent experiments performed in duplicate and expressed as mean ± SD. Unpaired t-test was used for statistical analysis.


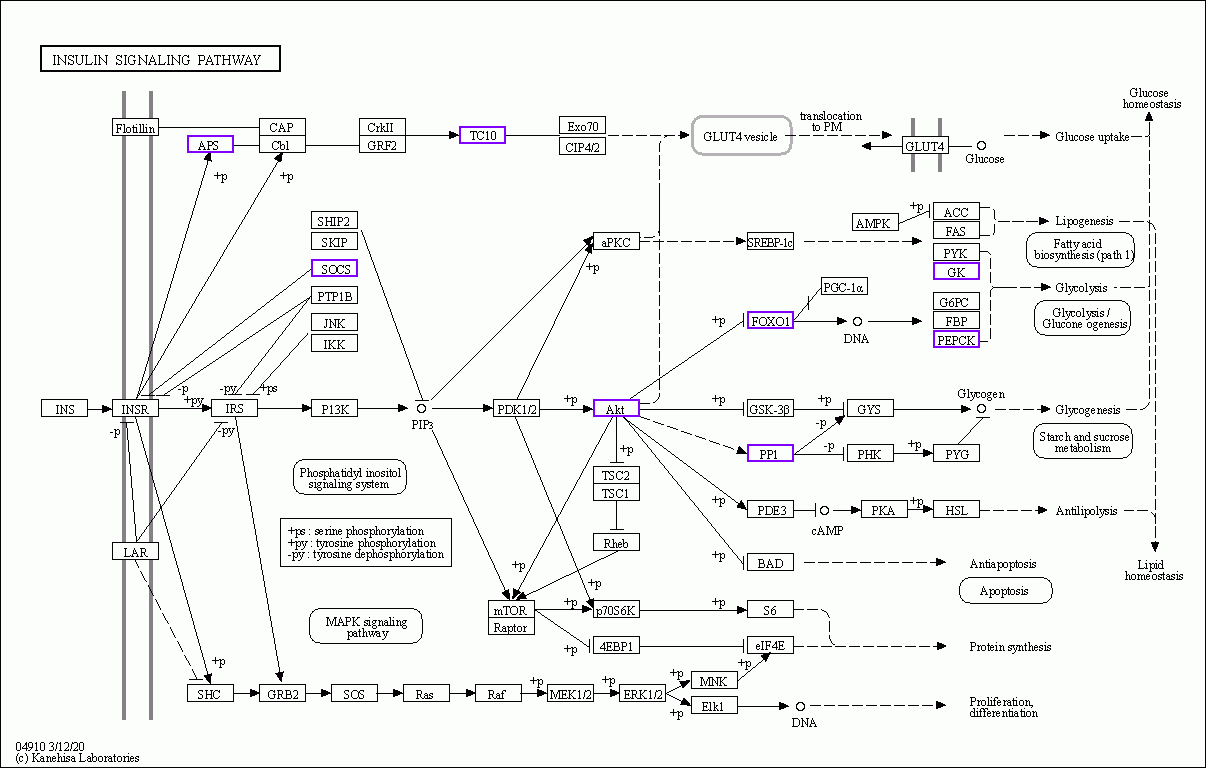


**Figure S2.** Schematic representation of the insulin signaling pathway (KEGG pathway map 04910) following 144 hours of exposure to TPN 1%. Differentially expressed genes are indicated in purple.

**
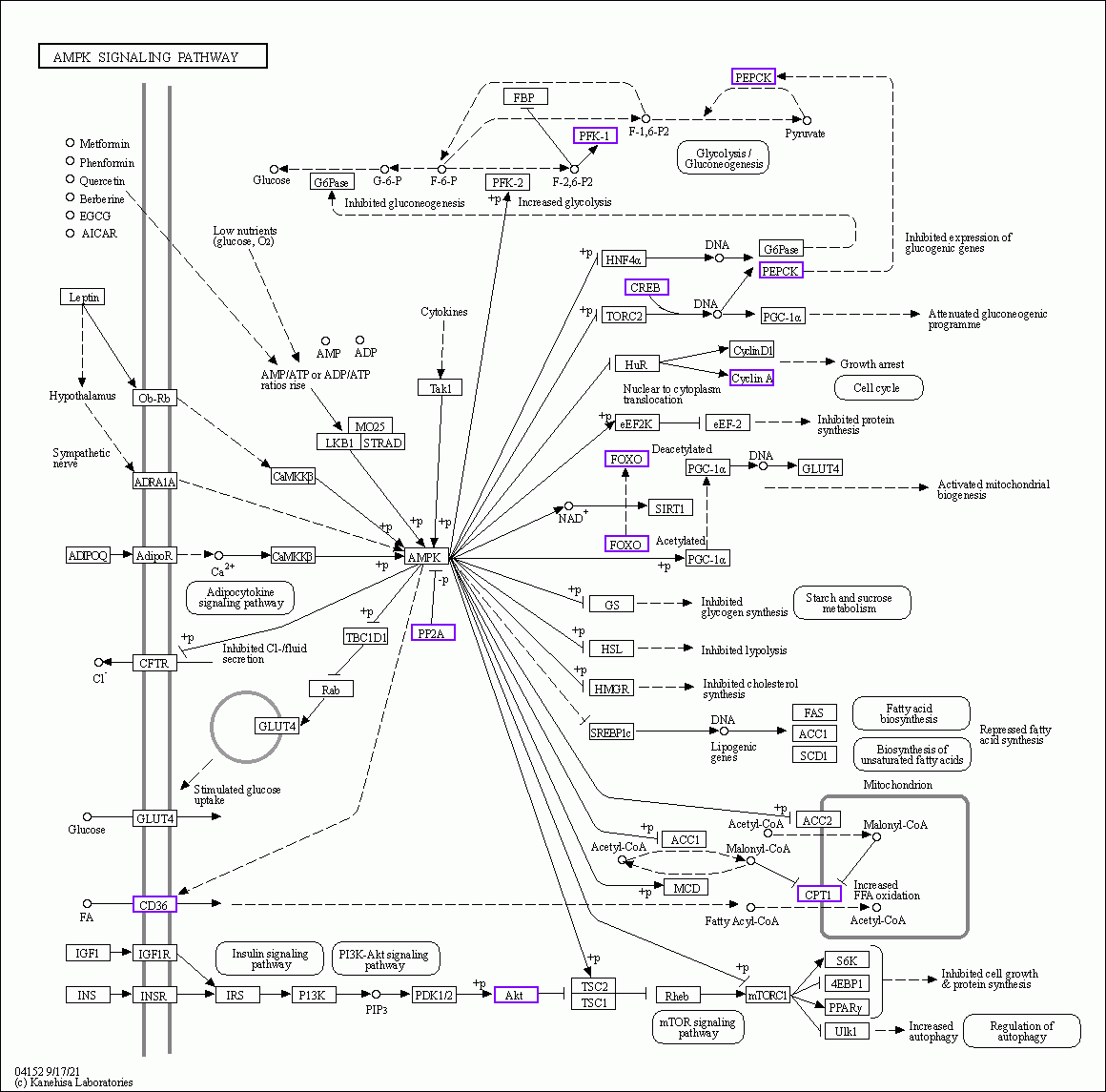
**

**Figure S3.** Schematic representation of the AMPK signaling pathway (KEGG pathway map 04152) following 144 hours of exposure to TPN 1%. Differentially expressed genes are indicated in purple.


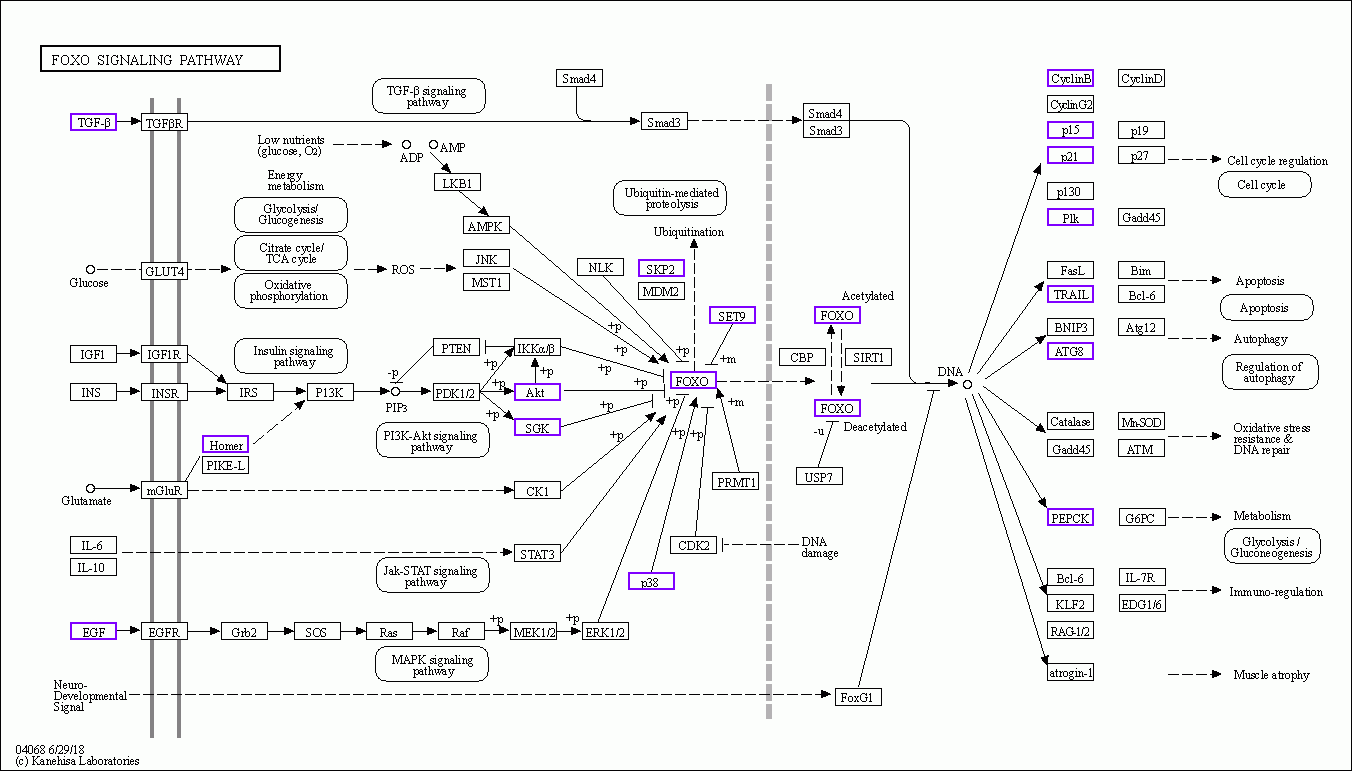


**Figure S4.** Schematic representation of the FoxO signaling pathway (KEGG pathway map 04068) following 144 hours of exposure to TPN 1%. Differentially expressed genes are indicated in purple.


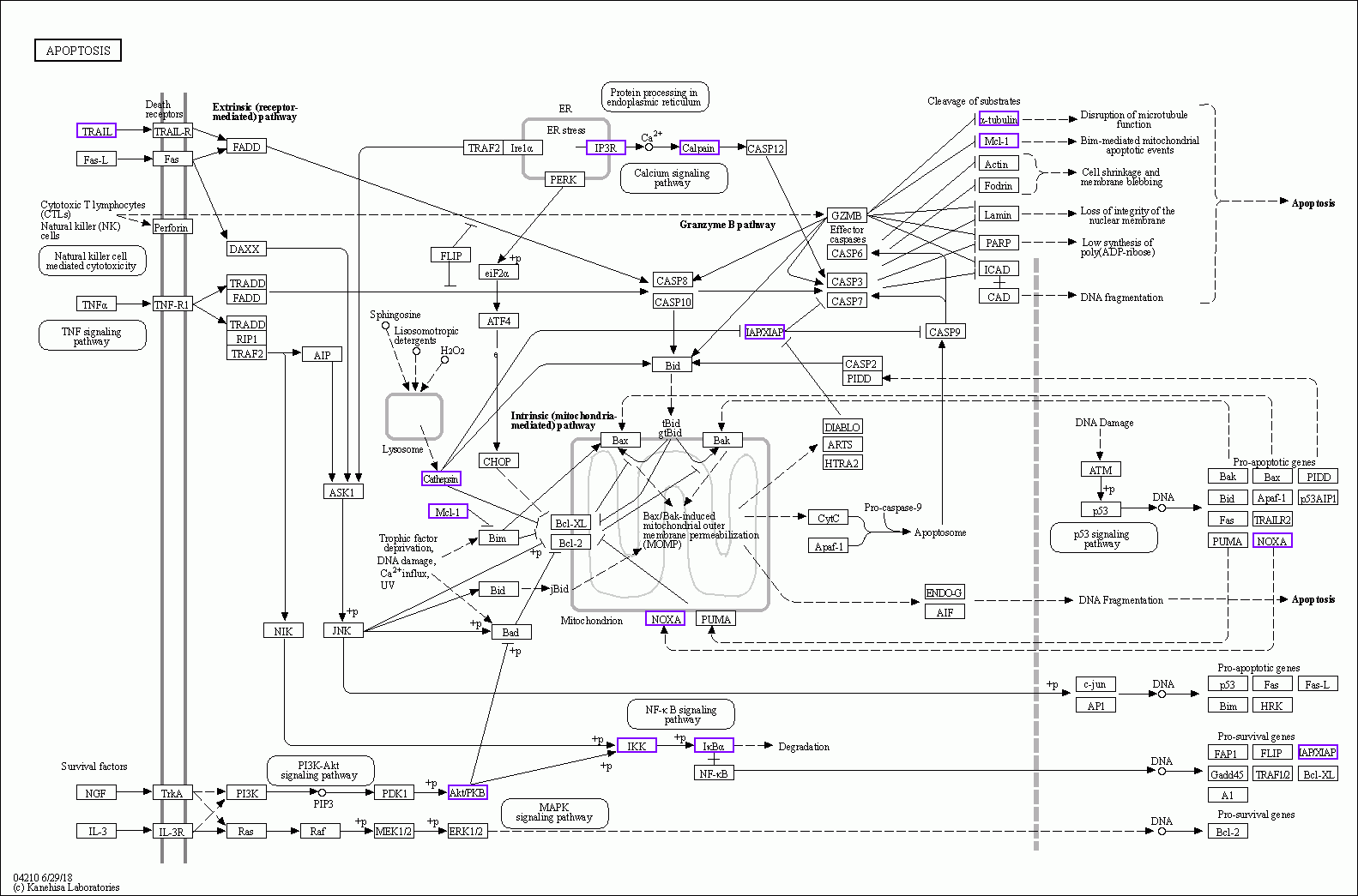


**Figure S5.** Schematic representation of apoptosis pathway (KEGG pathway map 04210) following 144 hours of exposure to TPN 1%. Differentially expressed genes are indicated in purple.


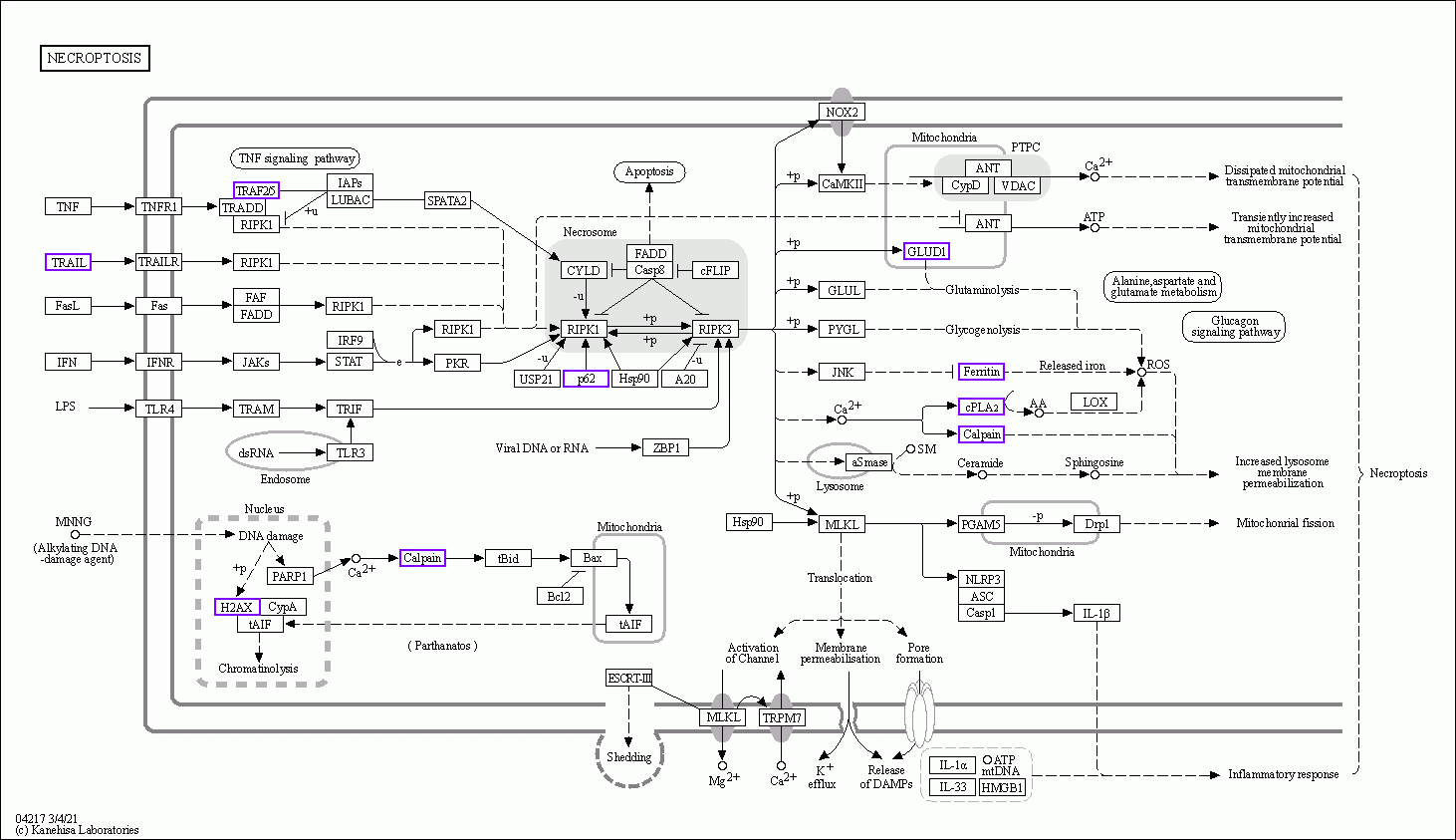


**Figure S6.** Schematic representation of necroptosis pathway (KEGG pathway map 04217) following 144 hours of exposure to TPN 1%. Differentially expressed genes are indicated in purple.


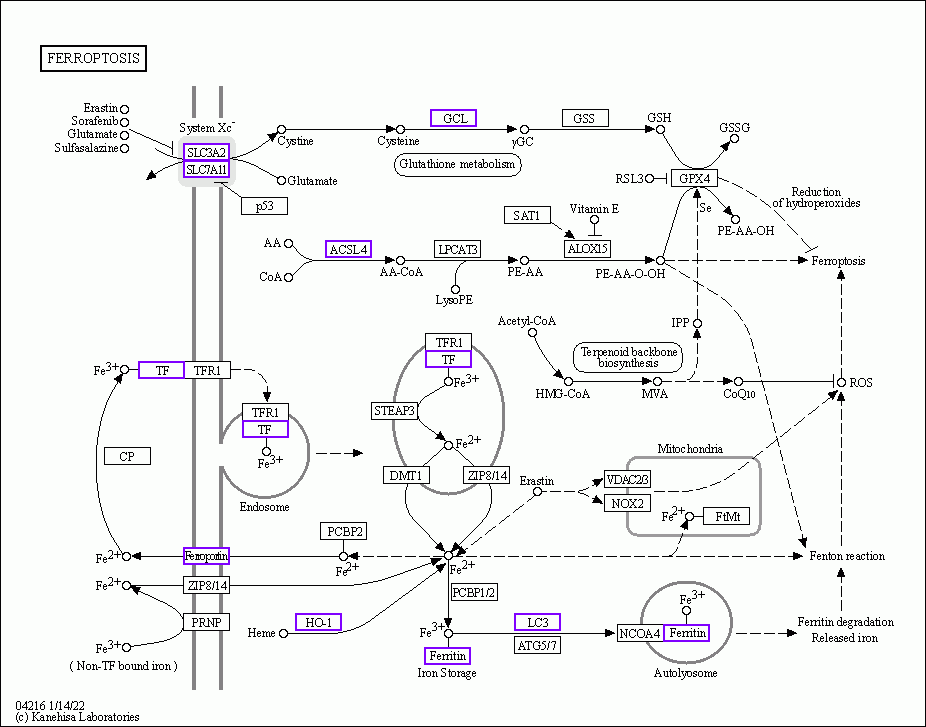


**Figure S7.** Schematic representation of ferroptosis pathway (KEGG pathway map 04216) following 144 hours of exposure to TPN 1%. Differentially expressed genes are indicated in purple.


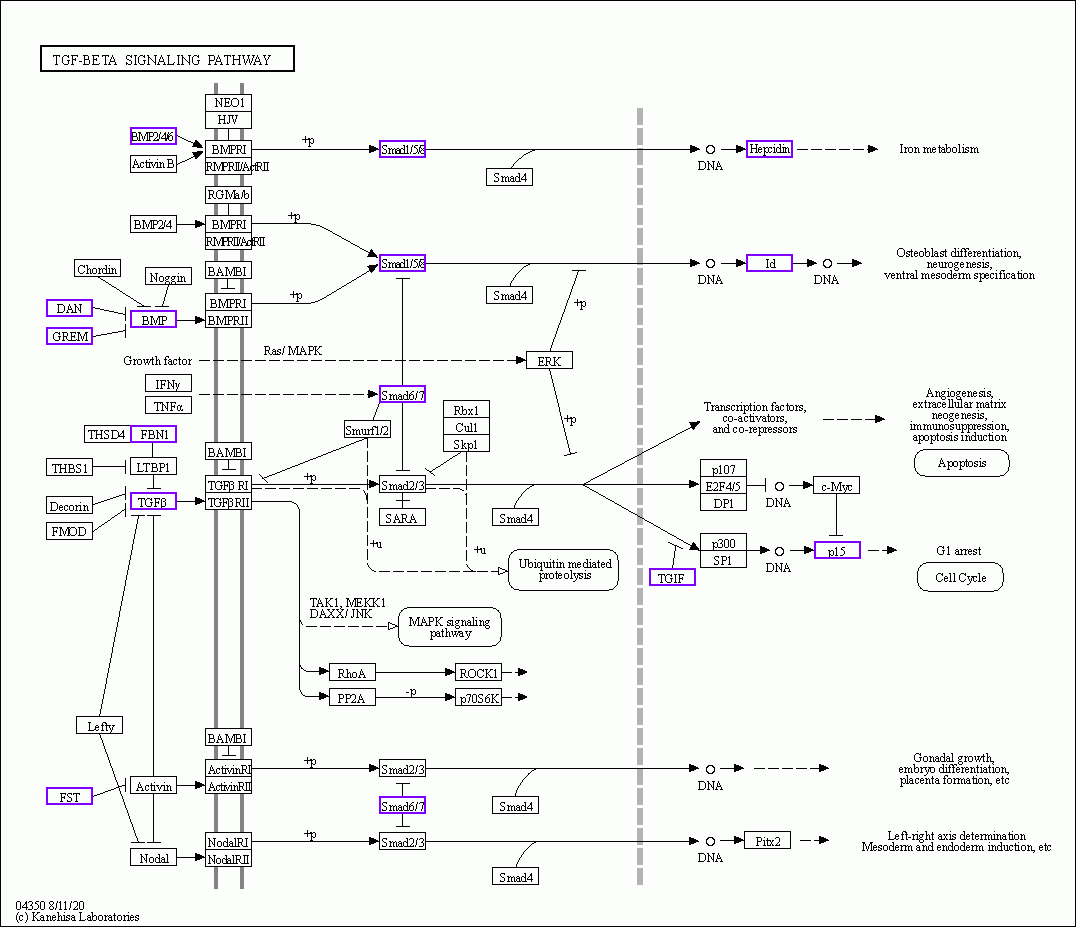


**Figure S8.** Schematic representation of the TGF-β signaling pathway (KEGG pathway map 04350) following 144 hours of exposure to TPN 1%. Differentially expressed genes are indicated in purple.


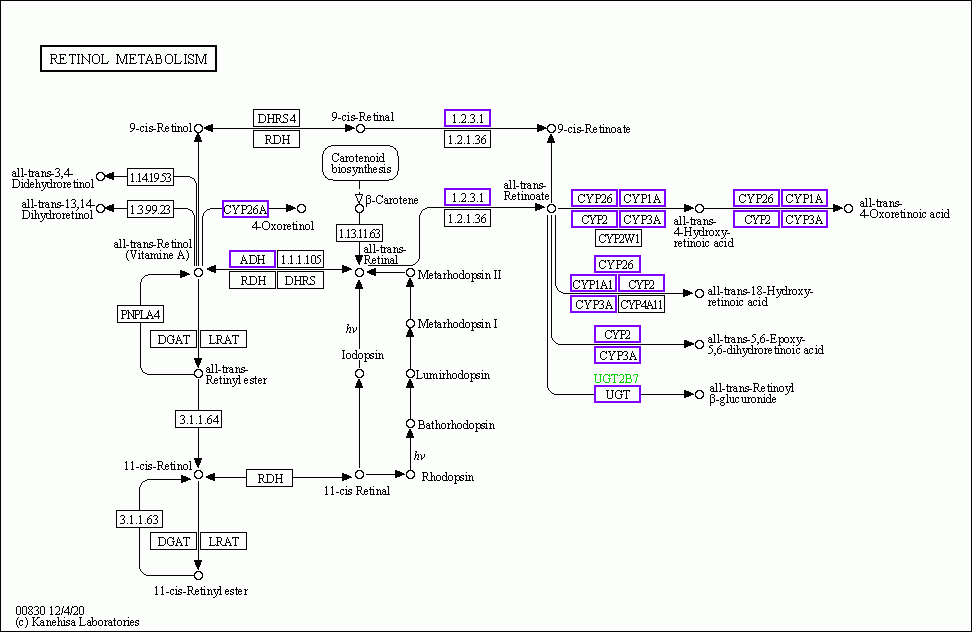


**Figure S9.** Schematic representation of retinol metabolism (KEGG pathway map 00830) following 144 hours of exposure to TPN 1%. Differentially expressed genes are indicated in purple.


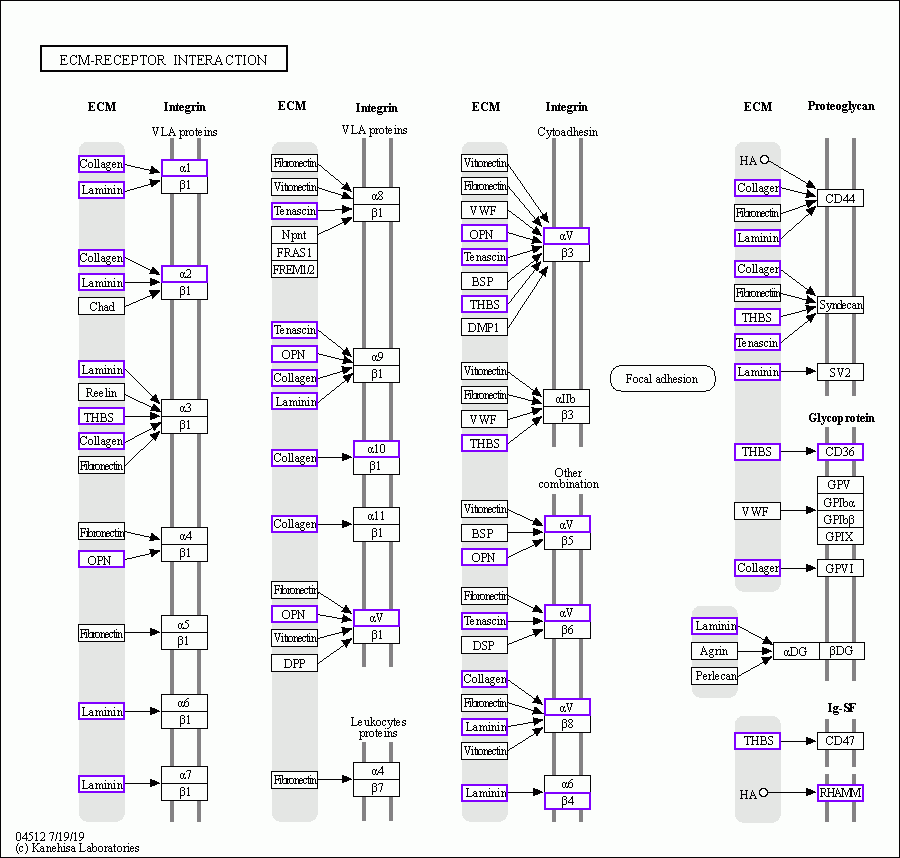


**Figure S10.** Schematic representation of extracellular matrix (ECM)-receptors interactions (KEGG pathway map 04512) following 144 hours of exposure to TPN 1%. Differentially expressed genes are indicated in purple.


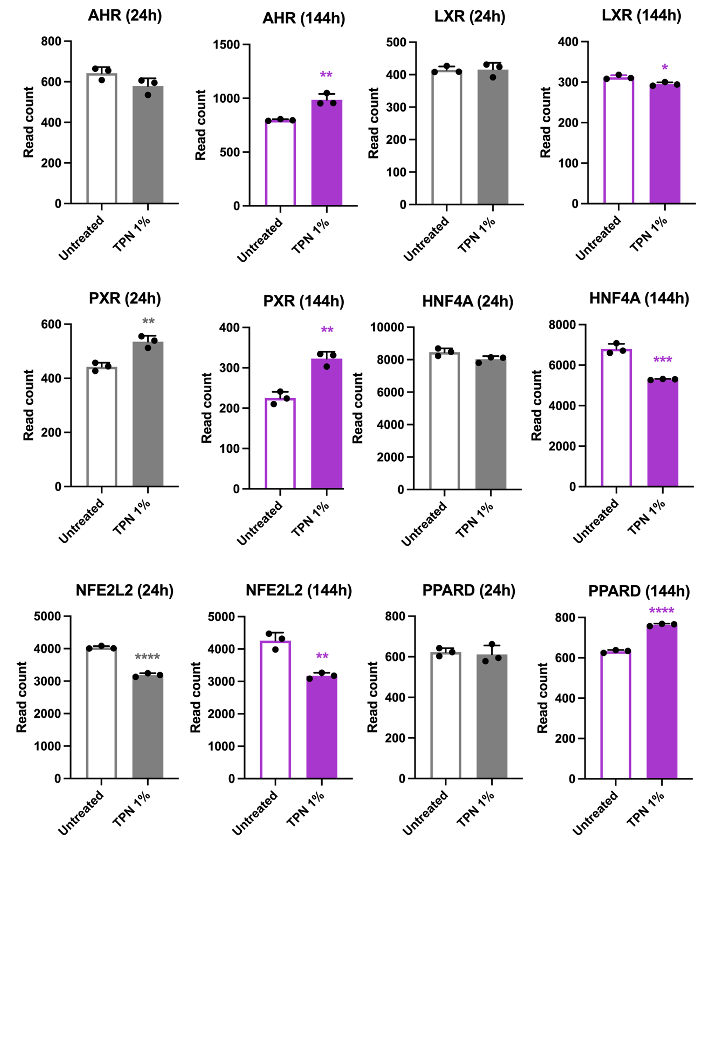
**Figure S11.** The mRNA levels of nuclear receptors and transcription factors involved in molecular initiating events of steatosis, after 24 hours or 144 hours of exposure to TPN 1%. AHR - aryl hydrocarbon receptor; LXR - liver X receptor; PXR - pregnane X receptor; HNF4A - hepatocyte nuclear factor 4 alpha; NFE2L2 - nuclear factor erythroid 2-related factor 2; PPARD – peroxisome proliferator-activated receptor delta. Read count values derived from RNA sequencing analysis from 3 independent experiments are expressed as mean ± SD. Unpaired t-test was used for statistical analysis; * p < 0.05, ** p < 0.01, *** p < 0.001, **** p < 0.0001.
